# Cardiothoracic Imaging for Outcome Prediction in Chronic Thromboembolic Pulmonary Hypertension after Pulmonary Endarterectomy or Balloon Pulmonary Angioplasty: A Scoping Review

**DOI:** 10.3390/jcm13175045

**Published:** 2024-08-26

**Authors:** Mikail Malik, Shamir Malik, Gauri R. Karur, Sebastian Mafeld, Marc de Perrot, Micheal C. McInnis

**Affiliations:** 1 Temerty Faculty of Medicine, University of Toronto, Toronto, ON M5S 1A8, Canada; mikail.malik@mail.utoronto.ca (M.M.);; 2 University Medical Imaging Toronto, Toronto General Hospital, Toronto, ON M5G 2C4, Canada; 3 Division of Cardiothoracic Imaging, Department of Medical Imaging, University of Toronto, Toronto, ON M5T 1W7, Canada; 4 Division of Interventional Radiology, Department of Medical Imaging, University of Toronto, Toronto, ON M5T 1W7, Canada; 5 Division of Thoracic Surgery, Department of Surgery, Toronto General Hospital, Toronto, ON M5G 2C4, Canada

**Keywords:** chronic thromboembolic pulmonary hypertension, cardiothoracic imaging, computed tomography, magnetic resonance imaging, pulmonary endarterectomy, balloon angioplasty, predictors of outcome

## Abstract

There has been a rapid expansion in centers performing balloon pulmonary angioplasty (BPA) and pulmonary thromboendarterectomy (PTE) for chronic thromboembolic pulmonary hypertension (CTEPH). The purpose of this scoping review was to identify cardiothoracic imaging predictors of outcomes and to identify gaps to address in future work. A scoping review was conducted using the framework outlined by Arksey and O’Malley and Levac et al. in MEDLINE and EMBASE. The study protocol was preregistered in OSF Registries and performed according to the Preferred Reporting Items for Systematic Reviews and Meta-Analyses for Scoping Reviews (PRISMA-ScR) guidelines. There were 1117 identified studies, including 48 involving pulmonary thromboendarterectomy (n = 25) and balloon pulmonary angioplasty (n = 23). CT was the most common preoperative imaging modality used (n = 21) and CT level of disease was the most reported imaging predictor of outcomes for pulmonary thromboendarterectomy. Although must studies evaluated hemodynamic improvements, imaging was of additional use in predicting clinically significant procedural complications after balloon pulmonary angioplasty, as well as mortality and long-term outcome after pulmonary endarterectomy. Predictors reported in MRI and digital subtraction angiography were less commonly reported and warrant multicenter validation. Cardiothoracic imaging may predict clinically significant outcomes after balloon pulmonary angioplasty and pulmonary thromboendarterectomy. Radiologists involved in the assessment of CTEPH patients should be aware of key predictors and future investigations could focus on multicenter validation and new technologies.

## 1. Introduction

Chronic thromboembolic pulmonary hypertension (CTEPH) is an underrecognized complication of acute pulmonary embolism, which is estimated to occur in around 3% of survivors, with significant morbidity and mortality if left untreated [1]. Much of the radiologic literature has focused on the diagnosis of CTEPH with an emphasis on ventilation perfusion (VQ) scintigraphy as a highly sensitive screening test now embedded in guidelines for the workup of pulmonary hypertension [2]. However, there have been advances in the imaging diagnosis of CTEPH, including the integration of single-photon emission computed tomography (SPECT), digital subtraction angiography (DSA), computed tomography pulmonary angiography (CTPA), and dual-energy CT (DECT). At expert centers, CTPA now achieves a sensitivity and specificity comparable to VQ scintigraphy with regard to the diagnosis of CTEPH [3,4].

Alongside these advances in imaging tools came substantial advancements in the treatment of CTEPH, which are now leading to substantial recovery in a large proportion of patients. Pulmonary thromboendarterectomy (PTE) is a surgical procedure that removes chronic thromboemboli, improving blood flow and normalizing pressures, and achieving excellent long-term outcomes with low perioperative mortality at experienced centers [5]. Balloon pulmonary angioplasty (BPA) is a minimally invasive procedure that disrupts the thromboemboli to improve blood flow and potentially normalize pressures [6]. Despite these promising treatments, many patients experience residual or recurrent pulmonary hypertension after treatment and may require medical therapies or repeated interventional procedures necessitating additional imaging to guide therapy [7].

Now that effective intervention is mainstream, it is necessary that imaging tests that patients undergo provide information about the accessibility of the chronic pulmonary emboli and, ideally, the imaging test of choice will offer novel information for predicting patients’ outcomes after their subsequent treatment. To date, there is little available literature summarizing these imaging predictors of outcomes after treatment in CTEPH. Therefore, the purpose of this scoping review is to identify cardiothoracic imaging predictors of outcomes in patients with CTEPH who undergo PTE or BPA.

## 2. Materials and Methods

### 2.1. Search Strategy

This retrospective scoping review was conducted using the frameworks outlined by Arksey and O’Malley and Levac et al. [8,9]. The search strategy was developed with the assistance of librarians and involved two databases, plus gray literature searches following the Preferred Reporting Items for Systematic Reviews and Meta-Analyses for Scoping Reviews (PRISMA-ScR) [10]. A search was performed to identify if previous systematic or scoping reviews with similar research questions have been conducted. Six systematic reviews were identified and none had significant overlap with the proposed research question (Supplementary Table S1). Two databases, including OVID MEDLINE (1946–present) and EMBASE (1947–present), were searched on the concepts of ‘thoracic imaging’, ‘CT’, ‘MRI’, ‘ventilation–perfusion ratio’, ‘digital subtraction angiography’, ‘CTEPH’, ‘endarterectomy’ or ‘balloon angioplasty’ from inception up to March 2023. The full search strategy is available in the Supplementary Material. Gray literature was searched to identify relevant non-indexed literature such as conference proceedings, research reports, doctoral dissertations, by querying non-indexed citations in OVID MEDLINE and searching the archived OpenGrey database for the terms ‘CTEPH’, ‘PTE’, ‘BPA’, and associated imaging modalities. There were no restrictions on the year of publication. A protocol outlining the proposed search strategy, eligibility criteria, data extraction, outcomes, and analysis of this scoping review was registered a priori on Open-Science Framework [11].

### 2.2. Eligibility Criteria

To be included, studies must have followed patients diagnosed with CTEPH who had undergone PTE or BPA, and the studies had to have been written in the English language. Patients were required to have undergone either computed tomography (CT) of the thorax, DSA, VQ scintigraphy, or magnetic resonance imaging (MRI) of the thorax. Included studies must have had imaging conducted prior to PTE or BPA and must have had an outcome measurement at any time interval after patients had undergone either PTE or BPA. Studies using only echocardiography or right heart catheterization for evaluation were excluded.

### 2.3. Data Extraction

Screening and data extraction were performed by two independent reviewers (M.M, S.M) with conflicts resolved by consensus or the decision of the third reviewer (M.C.M). Data extracted included the total number of participants, demographic features, procedure received, imaging modality used, purpose of imaging predictor, described imaging predictors and their associated outcomes, the strength of the relationship between predictor and outcome, and bibliometric data.

### 2.4. Defining Outcomes and Analysis

Outcomes were broadly categorized into three groups: 1. Changes in hemodynamics (i.e., an imaging feature predicts changes in left ventricular cardiac output.); 2. changes in performance capacity (an imaging feature predicts changes in functional class, that is, the severity of patient symptoms and daily activity limitations); and 3. morbidity and mortality (i.e., an imaging feature predicts procedural or all-cause mortality, or length of stay). Data analysis involved a quantitative descriptive analysis of studies and a qualitative thematic analysis of common imaging predictors and their applications in CTEPH following PTE or BPA.

## 3. Results

**Figure 1:**
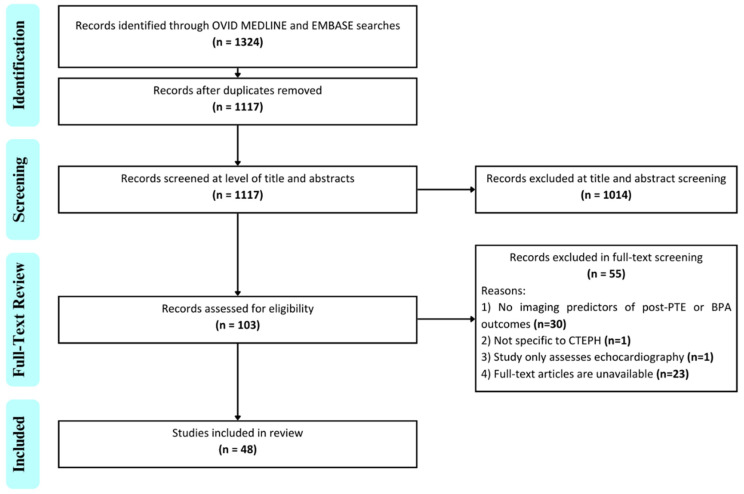
Preferred Reporting Items for Systematic Reviews and Meta-Analyses for Scoping Reviews (PRISMA-ScR) flow diagram of the search process and study selection. Twenty-three of the excluded articles were conference abstracts and missing full texts. Four of these abstracts were published in 2021 or later and may have been pending full publication at the time of the search. There were no technical issues or access problems. Abbreviations: PTE = pulmonary thromboendarterectomy; BPA = balloon pulmonary angioplasty; CTEPH = chronic thromboembolic pulmonary hypertension.

The database search yielded 1324 studies with 1117 studies remaining after duplicates were removed. There were 103 studies that underwent full-text review with 48 studies included after final review (Figure 1, Supplementary Tables S2 and S3). Detailed study characteristics are listed in Supplementary Table S4. Of the included studies, there was a near even distribution of those that investigated PTE (n = 25, 52%) and BPA (n = 23, 48%).

### 3.1. Bibliometric Findings

The rate of publication of imaging predictors for outcome prediction has increased over time commensurate with the expansion of CTEPH centers in the United States. Most included studies were published between 2018 and 2023 alone (n = 31, 65%), excluding studies that would be published in 2023 if the search was conducted at the end of the year. The median year of publication was 2018 for all studies, 2018 for PTE, and 2019 for BPA. There has been a notable growth in the publication of studies pertaining to BPA recently, whereas those pertaining to imaging predictors of post-PTE outcomes date back to 2000. While CT and MRI are the imaging modalities with similar volumes of literature describing imaging predictors of CTEPH, CT predictors have been steadily described for the past two decades, and MRI predictors have been more recently explored. Studies evaluating DSA predictors have become less common (Supplementary Figure S1). Country of origin, centers involved, and journals for the included studies are appended (Supplementary Tables S2, S3 and S5).

### 3.2. Outcomes Measured

The most common outcomes predicted post-PTE and BPA were changes in hemodynamics (n = 37, 77%), followed by measures of morbidity or mortality (n = 14, 29%), and changes in performance capacity (n = 10, 21%). The most common hemodynamic outcome measures in both PTE and BPA studies were changes in mean pulmonary artery pressure and pulmonary vascular resistance. Mortality was more commonly measured in PTE studies and procedural complications were more commonly measured in BPA studies. Changes in performance capacity were uncommonly measured in both BPA and PTE and those limited studies were restricted to changes in a 6 min walk test.

### 3.3. Imaging Modality

The most commonly investigated imaging modality was CT (n = 21, 44%) followed by MRI (n = 18, 38%) (Table 1). No included studies assessed VQ scintigraphy as a predictor of patient outcome. CT is a diverse technique, and the most reported acquisition was a conventional CTPA study with only two studies utilizing dual energy. Interestingly, several studies evaluated non-contrast CT. While lung subtraction iodine mapping is the subject of an ongoing clinical trial in the diagnosis of CTEPH, no reported studies utilizing lung subtraction iodine mapping were found [12].

The most common significant imaging predictor used in the CT studies were measures of the chronic thromboembolic distribution or proximity within the pulmonary arteries prior to PTE known as the CT level of disease (n = 4) (Table 2) [13,14,15,16]. This was followed by changes in whole lung perfusion blood volume (PBV) seen after BPA (n = 2), and measures of the pulmonary artery diameter before PTE (n = 1) or BPA (n = 1) (Table 2) [17,18,19,20,21].

The second most common imaging modality used was MRI, wherein cardiac MRI was the imaging technique used most (n = 14, 29%). MRI predictors are both numerous and diverse with 33 significant predictors identified across 18 studies, and most were only used in a single study. The most common significant MR imaging predictors were changes in the right ventricle end diastolic and end systolic volume indices (n = 2) after BPA (Table 3) [22,23].

Conventional angiography was not commonly investigated, with only four significant imaging predictors reported across eight studies. In contrast to CT and MRI, DSA imaging predictors were commonly assessed for outcomes pertaining to mortality or procedural complications rather than hemodynamic changes. The significant DSA imaging predictors identified were the presence of pouch or membrane segments prior to PTE (n = 1), perfusion status of subpleural spaces prior to PTE (n = 1), the presence of occlusive lesions after BPA (n = 1), and the presence of subtotal lesions before BPA (n = 1) (Table 4) [19,24,25,26].

Usage of SPECT was limited to two studies investigating BPA. The significant SPECT imaging predictors identified were the functional volume of the lung after BPA (n = 1), the increase in total uptake volume and the fractal dimension after BPA (both n = 1) (Table 5) [27,28].

**Figure 2:**
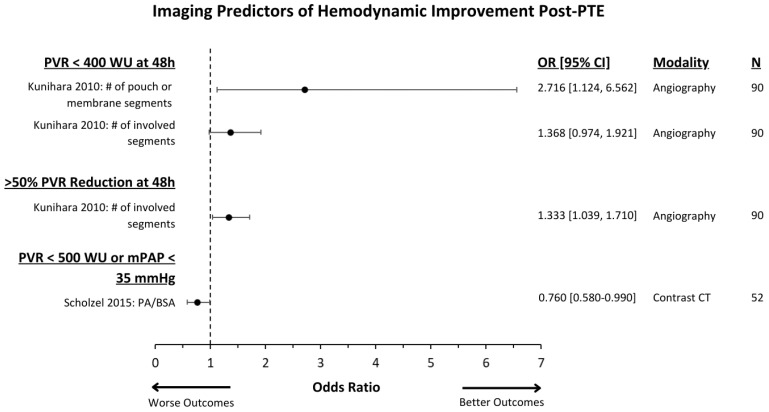
Imaging predictors of post-PTE hemodynamic outcomes reported with multivariate odds ratios and 95% CIs are presented [24,29]. “N” refers to the number of patients in each study. Abbreviations: PTE = pulmonary thromboendarterectomy; PVR = pulmonary vascular resistance; WU = Wood Units (dyn·s·cm^−5^); mPAP = mean pulmonary artery pressure; PA/BSA = pulmonary artery/body surface area; OR = odds ratio; CT = computed tomography.

**Figure 3:**
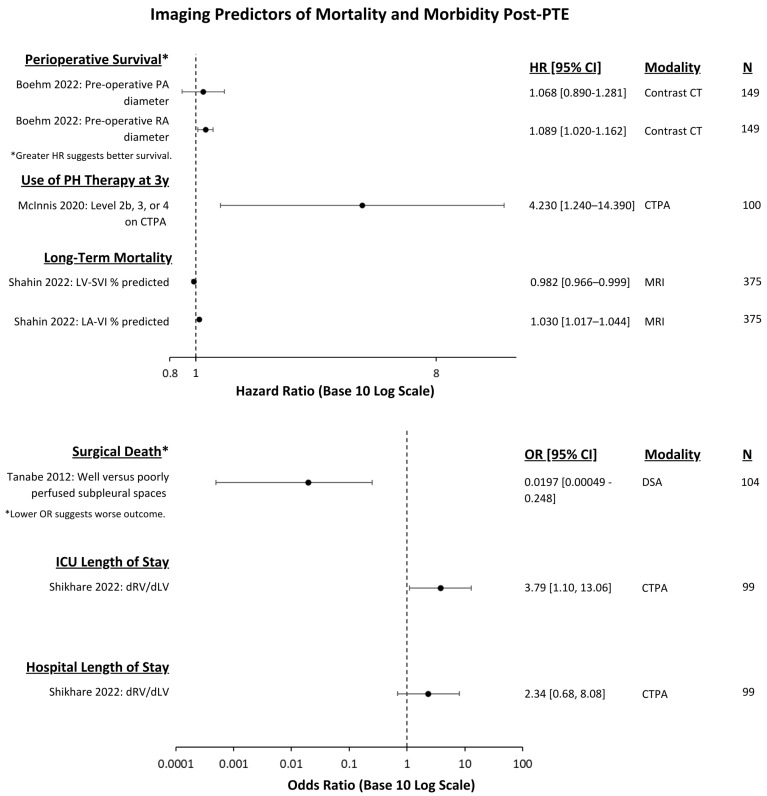
Imaging predictors of post-PTE mortality and morbidity outcomes that were reported with multivariate OR or HR with 95% CIs [13,20,25,30,31]. Hazard ratios and odds ratios are presented in separate forest plots. A base 10 logarithmic scale is used to facilitate wide confidence intervals. Use of PH therapy refers to patients requiring targeted medical therapy after PTE. Level 2b refers to disease in the basal trunk superior to segmental pulmonary artery, level 3 refers to disease starting in segmental pulmonary arteries within 1cm of origin, and level 4 refers to disease starting > 1 cm from origin of the segmental vessel and located mainly in the subsegmental vessel. “N” refers to the number of patients in each study. Abbreviations: PTE = pulmonary thromboendarterectomy, PA = pulmonary artery, RA = right atrium, PH = pulmonary hypertension, CTPA = computed tomography pulmonary angiogram, LV-SVI = left ventricular stroke volume index, LA-VI = left atrial volume index, dRV/dLV = right ventricle diameter/left ventricle diameter, MRI = magnetic resonance imaging, DSA = digital subtraction angiography, HR = hazard ratio, and OR = odds ratio.

**Figure 4:**
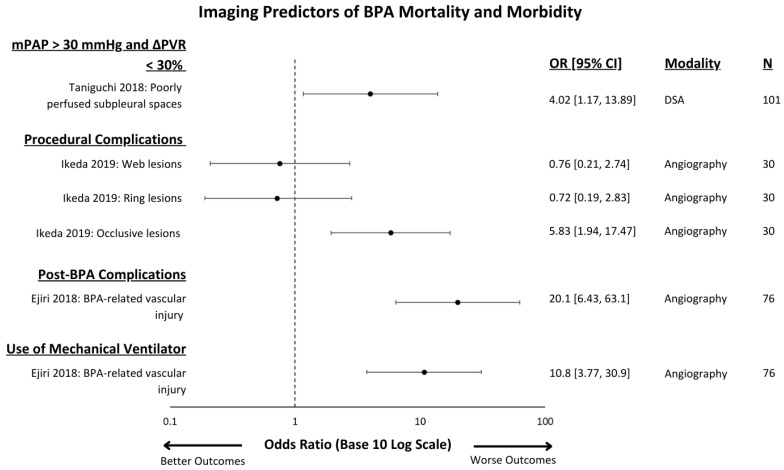
Imaging predictors of post-BPA outcomes that were reported with multivariate ORs and 95% CIs [19,32,33]. All predictors presented are angiographic findings. A base 10 logarithmic scale is used to facilitate wide confidence intervals. “N” refers to the number of patients in each study. Abbreviations: BPA = balloon pulmonary angioplasty, mPAP = mean pulmonary artery pressure, PVR = pulmonary vascular resistance, DSA = digital subtraction angiography, OR = odds ratio.

The effect of the imaging test and associated outcomes measures are demonstrated in Figure 2 and Figure 3 for PTE and in Figure 4 for BPA.

## 4. Discussion

We identified a relatively small number of studies assessing imaging predictors of outcomes in CTEPH interventions despite widespread use of imaging in the preoperative diagnosis of CTEPH. Although VQ scintigraphy and conventional angiography are considered the gold standard tests for screening and diagnosis, respectively, CT was the most commonly investigated imaging test. There was striking heterogeneity across studies in the variables in the imaging predictors assessed, with CT level of disease being the only measure determined to be significant in more than two studies [13,14,15,16]. This is likely because CT level of disease directly correlates with the well-established UCSD surgical level, which is crucial for assessing the extent of thromboembolic disease and surgical complexity. CT level of disease thus serves as a direct imaging correlate unlike other imaging predictors. As expected, the most common outcomes measured were changes in mean pulmonary artery pressure and pulmonary vascular resistance and we found a difference between PTE and BPA, with mortality more commonly measured in the former and procedural complications in the latter. Establishing a standardized set of outcomes would be valuable for improving the comparability of findings across studies. Implementing outcomes like the 6 min walk test would provide crucial insight into functional recovery following PTE or BPA. Since persistent pulmonary hypertension remains a concern post-procedure, tracking improvements in mPAP and PVR would also be essential. While mortality is important, it is relatively uncommon. The number of investigations is increasing over time, with a greater proportion of BPA work in recent years.

In assessing patients’ expected outcome after a procedure in CTEPH, the interventionist considers the severity of pulmonary hypertension, comorbidities and the amenability of the patient’s disease distribution to the proposed therapy among other factors. For example, disease in the main, lobar or proximal segmental pulmonary vasculature would be considered amenable to PTE whereas disease restricted to the distal segmental and subsegmental vasculature would be considered amenable to BPA, though this distinction is highly dependent on the level of operator expertise. This scoping review highlights areas where the interventionist can leverage imaging in determining outcome in addition to the clinical factors.

In the assessment for patients undergoing PTE, we found that CT is key in the preoperative prediction of patient outcome. Boehm et al. demonstrate that the ratio of the pulmonary artery to ascending aorta is associated with lower survival probability as it increases and correlates with worse pulmonary hypertension [20]. Shikhare et al. demonstrate that non-gated measures of the right to left ventricle ratio were easy to perform, correlated with severity of pulmonary hypertension, and that a higher ratio was associated with a longer length of stay in the intensive care unit [31]. McInnis et al. demonstrated the importance of CT in predicting the long-term prognosis of patients after PTE using the CT level of disease [13]. Analogous to the UCSD level, they found that disease restricted to the segmental and subsegmental vasculature was an independent risk factor for requiring postoperative pulmonary hypertension therapy.

In the assessment of patients undergoing BPA, CT and MRI were the most common imaging modalities explored for predicting treatment effect and patient outcomes. Koike et al. demonstrated that improvements in lung perfusion blood volume seen on dual-energy CT correlated with improvements in pulmonary arterial pressure, pulmonary vascular resistance, and 6 min walking distance [18]. Nishina et al. and Yamasaki et al. discovered correlations between reductions in right ventricular end systolic and end diastolic volume indices with reductions in pulmonary vascular resistance after BPA, highlighting the potential of cardiac MRI for non-invasive investigations of treatment effect [22,23]. Nishina et al. also demonstrated that the pre-BPA septal inversion ratio calculated using cardiac MRI was an independent predictor of pulmonary hypertension alleviation.

Several studies used DSA to explore imaging predictors of BPA failure and other complications. Taniguchi et al. showed that minimally perfused subpleural spaces on DSA prior to BPA are associated with persistent pulmonary hypertension and lesser reductions in pulmonary vascular resistance [32]. Ikeda et al. investigated lesion morphology with DSA and discovered that occlusive lesions that completely obstruct blood flow to distal vessels were the only morphology associated with higher risk of BPA-related procedural complications such as pulmonary bleeding or pulmonary artery dissections [19].

The use of MR imaging predictors for patient outcomes post-PTE or BPA is relatively novel and has seen increased usage within the last 5 years. Unlike CT imaging, MRI approaches do not involve ionizing radiation and allow comprehensive qualitative and quantitative cardiovascular assessment. Although several MR imaging techniques, such as MR lung perfusion and MR pulmonary angiography, have been studied in the context of diagnosing CTEPH, cardiac MRI was the most widely used technique among studies assessing imaging predictors of post-PTE and BPA outcomes [34,35]. Cardiac MRI is considered the reference standard for non-invasive measurements of right ventricular function and morphology, allowing for reproducible evaluation of ventricular remodeling via quantification of mass, volumes, and myocardial strain indices in CTEPH patients [36]. MRI also offers flow quantification using phase contrast analysis which can accurately measure metrics such as cardiac output and valvular regurgitant fractions [37]. Another advantage of cardiac MRI is the ability to identify fibrosis, which is associated with increased mortality in cardiomyopathy, through several techniques such as late gadolinium enhancement, and myocardial T1 and extracellular volume mapping (ECV) [38,39]. In the context of CTEPH, there is evidence that native T1 mapping and ECV values are associated with hemodynamic outcomes such as ejection fraction pre-operatively; however, there are no associations with these techniques and mortality [38,40].

Leong et al. demonstrated the ability of eleven different cardiac MRI feature-tracking strain assessment parameters, notably peak right atrial strain, to predict a high-risk status in patients undergoing PTE [41]. Kamada et al. utilized thoracic four-dimensional flow MRI and found significant changes in components of vortical flow in the pulmonary arteries following BPA [42]. However, changes in these imaging markers from before to after BPA did not significantly predict hemodynamic outcomes such as mean pulmonary artery pressure. Techniques such as feature tracking and 4D flow MRI do show promise for cardiac MRI, but they are limited as they are not routinely performed in practice. While attractive as a non-invasive test, the use of MRI is also inherently limited by the longer duration of the test, wait times, cost, and the availability of cardiovascular or thoracic imaging experts, which can vary between CTEPH centers [43].

Compared to conventional CTPA, novel imaging techniques such as DECT, LSIM, and photon-counting CT (PCCT) offer distinct advantages. DECT allows for the creation of perfusion-based images through differentiating tissue composition, improving the detection of pulmonary perfusion abnormalities. However, this requires specialized hardware. LSIM also allows for perfusion-based imaging but can be performed with a regular CT scanner, making it a more accessible option [12]. PCCT provides very high spatial resolution and improved image quality with reduced motion artifacts and radiation exposure to patients compared to conventional methods [44]. As PCCT is a newer technology, it is not yet widely available; however, further study would be of significant interest.

Cone-beam CT angiography is an emerging technique, with no studies meeting inclusion criteria. Compared to multidetector CT, cone-beam CT offers three-dimensional cross-sectional imaging of the pulmonary arteries with strong spatial resolution and contrast enhancement in real time, yet currently lacks large-scale data supporting its use [45]. Other CT technology, such as lung subtraction iodine mapping and photon-counting CT, would be logical areas of future study. However, we perceive that significant investigation using widely available technology would be of further interest.

Multicenter validation of imaging predictors is important to assess generalizability to different populations and better standardize care. CT level of disease was again the only predictor validated at more than two sites, including Toronto, Zurich (Eberhard et al.), Ottawa and Erlangen. McInnis et al. and Eberhard et al. both report moderate levels of interobserver agreement between reading radiologists for this measure, although agreement declines when scoring chronic thromboembolism at the segmental and subsegmental levels [13,14,46].

Optimal visualization methods for CTEPH involve a multimodal approach. All patients should be initially evaluated with a plain radiograph and a V/Q scan to assess for perfusion defects indicative of chronic thromboembolism. For all patients undergoing surgery, CTPA is essential for anatomical assessment, with only a minority of patients at our institute undergoing additional testing such as right heart catheterization or MRI. Although CT is vital for outcome prediction in CTEPH, it has its limitations. Effective interpretation requires local expertise, and the iodine-based contrast can pose issues for patients with severe allergies. CTPA also requires patient compliance with a breath hold, which can be challenging to perform. Despite the drawbacks, the widespread availability of CTPA remains a significant strength. We use MRI on a case-by-case basis, such as in those with a severe iodine contrast allergy or in cases where further interrogation of the right heart is required.

### 4.1. Future Directions

This scoping review has highlighted several gaps in aligning imaging studies with current clinical practice. Firstly, there is still a need for imaging predictors of clinically relevant outcomes such as length of stay and performance capacity (i.e., 6-min walk distance) following PTE or BPA. Having these non-invasive predictors of patient prognosis would be very useful for future CTEPH centers for stratifying patients by risk and monitoring their response to treatment. There also appear to be a lack of studies investigating predictors of patient outcomes in the long-term. The majority of included studies evaluated the relationship of imaging predictors with procedure success or failure at <6 months post-PTE or BPA. Understanding which imaging tests can predict long-term patient outcomes such as survival, persistent pulmonary hypertension, or usage of pulmonary hypertension medications at timepoints greater than 1 year would also help CTEPH centers with treatment planning and longitudinal management. Given the immense variety of reported imaging predictors employed by various CTEPH centers, it would also be greatly beneficial to develop standardized imaging predictors to allow for more comparable data between studies, evaluation of the strength of predictors, and potentially influence the development of guidelines in the future. Standardizing outcome measures would also be valuable. While hemodynamic outcomes like mPAP and PVR changes are commonly used and effective, functional outcomes such as 6MWT are not consistently applied across studies. The primary focus of future studies should be on establishing a standardized set of imaging predictors and outcome measures, with validation across multiple centers to ensure generalizability and reliability. Given the rapid evolution of imaging technology and increasing rate of publication for imaging predictors of outcome, it would be prudent to update this scoping review in 4 to 5 years. If there are sufficient data and standardization of imaging predictors and outcomes, transitioning to a systematic review and meta-analysis would be appropriate.

### 4.2. Limitations

There are several limitations of this scoping review. Firstly, there are still limited and heterogeneous data with limited overlap of the imaging predictors for patient outcomes following treatment of operable or inoperable CTEPH, making it more challenging to identify common predictors and trends in publication. Similarly, there are many different endpoints reported to be associated with an imaging predictor; however, only a few outcomes, such as mean pulmonary artery pressure and pulmonary vascular resistance, are consistently used between studies. Furthermore, the statistical approaches used to determine the strength of the imaging predictors between studies were also variable. Most studies reported Spearman correlation coefficients with *p*-values, others used odds ratios or hazard ratios with confidence intervals, and some employed receiver operator curves, making comparison between studies challenging. Another limitation of the study is that the search was restricted to articles written in the English language.

## 5. Conclusions

This scoping review provides an overview of the imaging predictors used in the literature to evaluate post-PTE and BPA outcomes in CTEPH by intervention and modality and a catalog of significant imaging predictors used by different CTEPH centers. We recommend further efforts to investigate imaging predictors with clinically relevant short- and long-term outcomes using novel technology, with a focus on establishing a standardized set of imaging predictors and outcomes validated across multiple centers.

## Figures and Tables

**Table 1:** Imaging tests used to predict outcome after pulmonary thromboendarterectomy or balloon pulmonary angioplasty.

Imaging Modality		Hemodynamics	Mortality and Complications	Performance Capacity
Computed Tomography	CTPA	(n = 7/48)	(n = 3/48)	(n = 1/48)
	Other contrast-enhanced CT	(n = 4/48)	(n = 2/48)	(n = 1/48)
	Non-contrast	(n = 1/48)	(n = 2/48)	-
	Dual energy	(n = 2/48)	-	(n = 2/48)
DSA		(n = 4/48)	(n = 5/48)	-
Magnetic resonance imaging		(n = 17/48)	(n = 3/48)	(n = 6/48)
SPECT		(n = 2/48)	-	-
V/Q scintigraphy		-	-	-

Summary of modalities with described imaging predictors for outcomes that pertain to hemodynamics, morbidity and complications, and performance capacity. The number of studies (out of the 48 included) that describe an imaging predictor for each outcome is indicated by “n”. Other contrast-enhanced CT techniques included: ECG—gated CT with contrast and HRCT with contrast. Abbreviations: CT—computed tomography; CTPA—CT pulmonary angiography; DSA—digital subtraction angiography; SPECT—single-photon emission computed tomography.

**Table 2:** Tabular summary of statistically significant CT imaging predictors of outcome.

	Hemodynamics			Complications			PC
Significant Imaging Predictors	mPAP	PVR	CO	Mortality	Procedural Complication	Length of Stay	6MWT
PULMONARY THROMBOENDARTERECTOMY							
CT level of disease (n = 4)	(n = 2)	(n = 3)			(n = 1)		
Interventricular septal curvature (n = 1)	(n = 1)	(n = 1)	(n = 1)				
Composite CT score (n = 1)	(n = 1)	(n = 1)					
Right to left ventricle ratio (n = 1)						(n = 1)	
Small vessel disease (n = 1)		(n = 1)					
PA to aortic diameter ratio (n = 1)				(n = 1)			
PA diameter (n = 1)				(n = 1)			
BALLOON PULMONARY ANGIOPLASTY							
Whole-lung PBV (n = 2)	(n = 1)	(n = 2)					(n = 2)
Long-axis pulmonary bleeding (n = 1)					(n = 1)		
Short-axis pulmonary bleeding (n = 1)					(n = 1)		
Pulmonary bleeding volume (n = 1)					(n = 1)		
PA diameter (n = 1)	(n = 1)						
RA diameter (n = 1)	(n = 1)						
Density changes in vascular centerlines (n = 1)	(n = 1)	(n = 1)					(n = 1)
Density changes in parenchymal areas (n = 1)	(n = 1)						

Significant CT imaging predictors are grouped by intervention, and outcomes are grouped into the most observed hemodynamic, mortality, length of stay, and performance capacity metrics. Disease presence in the main, left, right, and segmental pulmonary arteries, or CT level of disease, are the most cited imaging predictors following PTE. The number of studies (out of the 48 included) that describe a statistically significant imaging predictor is indicated by “n”. Abbreviations: PC—performance capacity; mPAP—mean pulmonary arterial pressure; PVR—pulmonary vascular resistance; CO—cardiac output; 6MWT—6 min walk test; CT—computed tomography; PA—pulmonary artery; PBV—perfusion blood volume; RA—right atrium.

**Table 3:** Tabular summary of statistically significant MR imaging predictors of outcome.

	Hemodynamics			Complications			PC
Significant Imaging Predictors	mPAP	PVR	CO	Mortality	Procedural Complication	Length of Stay	6MWT
PULMONARY THROMBOENDARTERECTOMY							
Minimum main PA area; minimum main PA volume; mean right PA centerline velocity; and maximum right PA spatial average helical flow index (each n = 1)	(n = 1)						
Right heart strain (n = 1)	(n = 1)	(n = 1)					
LV-SVI; LAVI (n = 1)				(n = 1)			
Δ Coaptation height (n = 1)			(n = 1)				
Max mean velocity through main PA (n = 1)	(n = 1)						(n = 1)
Average mean velocity in main PA (n = 1)							(n = 1)
Deceleration volume in main PA (n = 1)							(n = 1)
Median pPTT of whole lung; change in QDPpreful (each n = 1)	(n = 1)						
Change in PREFULq (n = 1)							(n = 1)
RV-EDV; RV-ESV; diastolic RV mass; systolic RV mass (each n = 1)	(n = 1)	(n = 1)					
Flow per beat; flow per minute (each n = 1)			(n = 1)				
BALLOON PULMONARY ANGIOPLASTY							
ΔRA max volume; ΔRA min volume; ΔRA ejection fraction; ΔRA peak longitudinal strain; Δ peak LSR; ΔRA early LSR (each n = 1)		(n = 1)					
Global longitudinal strain (n = 1)	(n = 1)	(n = 1)					
Apical area strain at RV apex (n = 1)		(n = 1)					
Pulmonary blood flow ratio (n = 1)			(n = 1)				
RV-EDVI (n = 2)		(n = 2)					
RV-ESVI (n = 2)		(n = 2)					
RV-EF (n = 1)		(n = 1)					
Septal inversion ratio (n = 1)		(n = 1)					

Significant MRI predictors are grouped by intervention, and outcomes are grouped into the most observed hemodynamic, mortality, length of stay, and performance capacity metrics. The number of studies (out of the 48 included) that describe a statistically significant imaging predictor is indicated by “n”. Abbreviations: PC—performance capacity; mPAP—mean pulmonary arterial pressure; PVR—pulmonary vascular resistance; CO—cardiac output; 6MWT—6 min walk test; PA—pulmonary artery; LV-SVI—left ventricular stroke volume index; LAVI—left atrial volume index; pPTT—pulmonary pulse wave transit time; QDPpreful—perfusion defect percentage based on phased-resolved functional lung of whole lung; PREFULq—phase-resolved functional lung MRI quantitative lung perfusion; RV-EDV—right ventricular end diastolic volume; RV-ESV—right ventricular end systolic volume; RV—right ventricular; LSR—longitudinal strain rate; RV-EDVI—right ventricle end diastolic volume index; RV-ESVI—right ventricle end systolic volume index; RV-EF—right ventricle ejection fraction.

**Table 4:** Tabular summary of statistically significant DSA imaging predictors of outcome.

	Hemodynamics			Complications			PC
Significant Imaging Predictors	mPAP	PVR	CO	Mortality	Procedural Complication	Length of Stay	6MWT
PULMONARY THROMBOENDARTERECTOMY							
Pouch or membrane segments (n = 1)		(n = 1)					
Subpleural perfusion (n = 1)		(n = 1)		(n = 1)			
BALLOON PULMONARY ANGIOPLASTY							
Occlusive lesions (n = 1)					(n = 1)		
Subtotal lesions (n = 1)					(n = 1)		

Significant DSA imaging predictors are grouped by intervention, and outcomes are grouped into the most observed hemodynamic, mortality, length of stay, and performance capacity metrics. The number of studies (out of the 48 included) that describe a statistically significant imaging predictor is indicated by “n”. Abbreviations: PC—performance capacity; mPAP—mean pulmonary arterial pressure; PVR—pulmonary vascular resistance; CO—cardiac output; 6MWT—6 min walk test.

**Table 5:** Tabular summary of statistically significant SPECT imaging predictors of outcome.

	Hemodynamics			Complications			PC
Significant Imaging Predictors	mPAP	PVR	CO	Mortality	Procedural Complication	Length of Stay	6MWT
BALLOON PULMONARY ANGIOPLASTY							
Functional volume of lung (n = 1)	(n = 1)						
Fractal dimension (n = 1)	(n = 1)						
Total uptake volume (n = 1)	(n = 1)						

Significant SPECT imaging predictors are grouped by intervention, and outcomes are grouped into the most observed hemodynamic, mortality, length of stay, and performance capacity metrics. The number of studies (out of the 48 included) that describe a statistically significant imaging predictor is indicated by “n”. Abbreviations: SPECT—single-photon emission computed tomography; PC—performance capacity; mPAP—mean pulmonary arterial pressure; PVR—pulmonary vascular resistance; CO—cardiac output; 6MWT—6 min walk test.

## Data Availability

No new data were created or analyzed in this study. Data sharing is not applicable to this article.

## Supplementary Materials

The following supporting information can be downloaded at https://www.mdpi.com/article/10.3390/jcm13175045/s1, Complete Search Strategy; Table S1: PubMed search prior to beginning review [4,6,47,48,49,50]; Table S2: Studies using imaging predictors for post-PTE outcomes [13,14,15,16,20,24,25,29,30,31,41,51,52,53,54,55,56,57,58,59,60,61,62,63,64]; Table S3: Studies using imaging predictors for post-BPA outcomes [17,18,19,20,21,22,23,26,27,28,32,33,42,65,66,67,68,69,70,71,72,73,74,75]; Table S4: Characteristics of included studies by intervention; Table S5: Country and Journals of Publication [13,14,15,16,17,18,19,20,21,22,23,24,25,26,27,28,29,30,31,32,33,41,42,51,52,53,54,55,56,57,58,59,60,61,62,63,64,65,66,67,68,69,70,71,72,73,74,75]; Figure S1: Temporal trend in imaging tests studied over time.
