# Supplementary material for: Cardiothoracic Imaging for Outcome Prediction in Chronic Thromboembolic Pulmonary Hypertension after Pulmonary Endarterectomy or Balloon Pulmonary Angioplasty: A Scoping Review
Source: J Clin Med. 2024 Aug 26;13(17):5045. doi: 10.3390/jcm13175045 (PMC11395896; doi:10.3390/jcm13175045)
Supplement: Supplementary file 1 [file jcm-13-05045-s001.zip › jcm-3148094-supplementary.pdf]

<u>Supplemental Material</u>

Search for Previous Scoping/Systematic Reviews:

PubMed Search: ((chronic thromboembolic pulmonary hypertension OR ctep*) AND ((scoping review[Title]) OR (systematic review[Title]))) AND imaging

Complete Search Strategy:

Ovid MEDLINE: Epub Ahead of Print, In-Process & Other Non-Indexed Citations, Ovid MEDLINE® Daily and Ovid MEDLINE® <1946-Present>

1 diagnostic imaging/ or exp radiography, thoracic/ or exp magnetic resonance imaging/ or tomography, x-ray/ or exp tomography, x-ray computed/ 1002747
2 Ventilation-Perfusion Ratio/ 5724
3 Angiography, Digital Subtraction/ 11407
4 (imaging adj3 (diagnostic or thoracic or magnetic resonance)).tw,kf. 327014
5 (MRI or CT).tw,kf. 681386
6 (tomograph* adj3 (x ray or xray or computed)).tw,kf. 345249
7 (digital subtraction angiograph* or DSA or Ventilation-Perfusion Ratio* or VQ or thoracic radiograph*).tw,kf. 22079
8 1 or 2 or 3 or 4 or 5 or 6 or 7 1471029
9 Endarterectomy/ 7118
10 Angioplasty, Balloon/ 18683
11 (endarterectom* or thromboendarterectom*).tw,kf. 17542
12 (angioplast* adj3 (balloon* or pulmonary)).tw,kf. 11661
13 9 or 10 or 11 or 12 44933
14 hypertension, pulmonary/ or pulmonary arterial hypertension/ or pulmonary embolism/ 79713
15 (pulmonary adj3 (hypertension* or embolism* or thromboembolism*)).tw,kf. 99616
16 (CTEPH or CTEPD).tw,kf. 1794
17 14 or 15 or 16 121014
18 8 and 13 and 17 **455**

Embase Classic+Embase <1947 to 2023 March 09>

1 exp diagnostic imaging/ 251655
2 exp thorax radiography/ 235819
3 exp nuclear magnetic resonance imaging/ 1206688
4 exp x-ray tomography/ or exp computed tomography pulmonary angiography/ or exp low-dose computed tomography/ or exp x-ray computed tomography/ 102059
5 exp lung scintiscanning/ 10780
6 exp lung ventilation perfusion ratio/ 7211
7 exp digital subtraction angiography/ 27623
8 (imaging adj3 (diagnostic or thoracic or magnetic resonance)).tw,kf. 437675
9 (MRI or CT).tw,kf. 1181414
10 (digital subtraction angiograph* or DSA or Ventilation-Perfusion Ratio* or VQ or thoracic radiograph*).tw,kf. 32513
11 1 or 2 or 3 or 4 or 5 or 6 or 7 or 8 or 9 or 10 2272236
12 exp pulmonary endarterectomy/ or exp endarterectomy/ 32482
13 exp angioplasty/ 102394
14 (endarterectom* or thromboendarterectom*).tw,kf. 26166
15 (angioplast* adj3 (balloon* or pulmonary)).tw,kf. 17716
16 exp percutaneous transluminal angioplasty/ 34627
17 12 or 13 or 14 or 15 or 16 132937
18 exp chronic thromboembolic pulmonary hypertension/ 5175
19 (CTEPH or CTEPD).tw,kf. 3847
20 18 or 19 5825
21 11 and 17 and 20 **868**

*1 article identified through reference searching.

Result #	Citation	Overlap with Proposed Review
1	Kennedy MK, Kennedy SA, Tan KT, de Perrot M, Bassett P, McInnis MC, Thenganatt J, Donahoe L, Granton J, Mafeld S. Balloon Pulmonary Angioplasty for Chronic Thromboembolic Pulmonary Hypertension: A Systematic Review and Meta-analysis. Cardiovasc Intervent Radiol. 2023 Jan;46(1):5-18. Doi: 10.1007/s00270-022-03323-8. Epub 2022 Dec 6. PMID: 36474104.	Evaluates post-BPA outcomes in CTEPH patients, but does not review imaging predictors for outcomes.<br>No major overlap.
2	Lambert L, Michalek P, Burgetova A. The diagnostic performance of CT pulmonary angiography in the detection of chronic thromboembolic pulmonary hypertension-systematic review and meta-analysis. Eur Radiol. 2022 Nov;32(11):7927-7935. doi: 10.1007/s00330-022-08804-5. Epub 2022 Apr 28. PMID: 35482124.	Evaluates ability of CTPA in diagnosis of CTEPH, not predictive ability for post-BPA or post-PTE outcomes.<br>No major overlap.
3	Chen Y, Li F, Luo J, Chen J, Luo P, Li J. Comparative Efficacy and Safety of Targeted Therapies for Chronic Thromboembolic Pulmonary Hypertension: A Systematic Review and Network Meta-Analysis. Can Respir J. 2021 Sep 1;2021:1626971. doi: 10.1155/2021/1626971. PMID: 34512819; PMCID: PMC8426079.	Evaluates efficacy of pharmacological CTEPH treatments.<br>No major overlap.
4	Dong C, Zhou M, Liu D, Long X, Guo T, Kong X. Diagnostic accuracy of computed tomography for chronic thromboembolic pulmonary hypertension: a systematic review and meta-analysis. PLoS One. 2015 Apr 29;10(4):e0126985. Doi: 10.1371/journal.pone.0126985. PMID: 25923810; PMCID: PMC4414539.	Evaluates ability of CT imaging techniques for diagnosis of CTEPH, not predictive ability for post-BPA or post-PTE outcomes.<br>No major overlap.
5	Li W, Yang T, Quan RL, Chen XX, An J, Zhao ZH, Liu ZH, Xiong CM, He JG, Gu Q. Balloon pulmonary angioplasty reverse right ventricular remodelling and dysfunction in patients with inoperable chronic thromboembolic pulmonary hypertension: a systematic review and meta-analysis. Eur Radiol. 2021 Jun;31(6):3898-3908. doi: 10.1007/s00330-020-07481-6. Epub 2020 Nov 17. PMID: 33201287.	Evaluates changes in RV function post-BPA using cardiac MRI and echocardiography, but does not review imaging predictors for outcomes.<br>No major overlap.
6	Jorge E, Baptista R, Calisto J, Faria H, Monteiro P, Pan M, Pêgo M. Optical coherence tomography of the pulmonary arteries: A systematic review. J Cardiol. 2016 Jan;67(1):6-14. doi: 10.1016/j.jjcc.2015.09.024. Epub 2015 Nov 10. PMID: 26572955.	Reviews applications of optical coherence tomography to the pulmonary arteries. Does not include imaging predictors for outcomes post BPA or PTE.<br>No major overlap.

Supplementary Table S1: A search was conducted on PubMed prior to beginning the review to evaluate the literature for overlap [4,6,47–50].

First Author	Centers	Imaging Technique	Explored Imaging Predictors	Hemodynamic Outcomes Predicted	Mortality and Morbidity Outcomes Predicted	Performance Capacity Outcomes Predicted
Studies using CT Imaging Predictors						
Liu	China-Japan Friendship Hospital	CTPA	*Radiomics signature of epicardial adipose tissue (5 features)*	N/A	*Post-operative atrial fibrillation*	N/A
Shikhare	Khoo Teck Puat Hospital, JDMI University of Toronto	CTPA	*dRV/dLV ratio*	N/A	Procedural mortality; *cardiopulmonary bypass time, cross-clamp time; circulatory arrest time; time in ICU;* length of stay in hospital	N/A
Eberhard	University Hospital Zurich, JDMI University of Toronto	CTPA	*"Level 1" disease of main/left/right PA, "Level 2a"disease in any lobar vessel, "Level 2b" disease in basal trunk superior to segmental PA, "Level 3" disease starting in segmental pulmonary arteries within 1cm of*	*mPAP, PVR,* TRPG, sPAP	N/A	N/A

			origin; # of lung segments with perfusion effects; overall perfusion defect score			
Niznansky	General University Hospital in Prague	CTPA	Main PA diameter; *Ao/PA diameter ratio*; *DAo/PA diameter ratio*; PA/BSA ratio; *PA/BMI ratio*	*1-year PASP; 2-year PASP*; 3-year PASP	N/A	N/A
McInnis	JDMI University of Toronto	CTPA	*"Level 1" disease of main/left/right PA, "Level 2a"disease in any lobar vessel, "Level 2b" disease in basal trunk superior to segmental PA, "Level 3" disease starting in segmental pulmonary arteries within 1cm of origin, "Level 4" disease starting >1cm from origin of of segmental vessel and located mainly in subsegmental vessel*	N/A	*Jamieson Type 3 Disease at surgery; use of pulmonary hypertension therapy after PEA; duration of circulatory arrest; d*uration of hospital stay; duration of intubation days	N/A

Ruigrok	VU University Medical Center	CTPA	Number of abnormal arteries remaining after PTE	mPAP; PVR	N/A	N/A
Saito	Chiba Medical Center	Contrast-enhanced ECG-gated CT	*Change in Systolic IVSC, Change in Diastolic IVSC*	*mPAP, PVR, CO, sPAP, dPAP,* CI	N/A	N/A
Leone	Sant'Orsola-Malpighi Hospital	CTPA	*CT-score (calculated from disease distribution, main PA diameter, mosaic perfusion pattern, and tricuspid regurgitation)*; *mosaic perfusion degree; main PA diameter*; tricuspid regurgitation; *unilateral/bilateral disease*; bronchial artery hypetrophy	*mPAP, PVR*	N/A	N/A
Shimizu	Chiba Medical Center	CTA	Total cross-sectional area of bronchial arteries	mPAP, PVR, CI	N/A	Change in PaO2; change in AaDO2
Oikonomou	Ottawa Hospital, Ottawa Heart Institute	Contrast-enhanced HRCT	*Presence of disease in central arteries; presence of disease in segmental arteries*; small	*mPAP, PVR*	N/A	N/A

			vessel factor, non-small vessel factor; sum of extent of mosaic perfusion pattern in areas with normal and abnormal arteries			
Boehm	Medical University of Vienna	Contrast-enhanced CT	*PA/Ao diameter ratio;* PA diameter; RA diameter	N/A	*30-day survival;* perioperative survival	N/A
Scholzel	St Antonius Hospital, University Hospitals Leuven	Contrast-enhanced MDCT	Ao diameter; *PA diameter*; PA/Ao diameter ratio; *PA/BSA ratio*	*Achievement of mPAP < 35mmHg or PVR < 500*	N/A	N/A
Heinrich	University Hospital of Saarland	Contast-enhanced CT	Main PA diameter; PA/Ao diameter ratio; *perfusion score; central thrombi score; scar score; combined perfusion and central thrombi score*	*PVR*	N/A	N/A
Bergin	University of California Health	CTA	*Small vessel disease*; central disease; segmental disease	*PVR*	N/A	N/A
Studies using Angiography Predictors						
Tanabe	Chiba Medical Center	DSA	*Well-perfused subpleural perfusion spaces (1+ segment well-*	*PVR*	*Surgical death*	N/A

			perfused) versus poor subpleural perfusion (all segments minimally or not perfused); central disease score			
Kunihara	University Hospital of Saarland	Conventional angiography	*Number of involved segments; number of pouch or membrane segments*	*PVR, 50% PVR reduction at 1-day*	N/A	N/A
Studies using MRI Predictors						
Dong	Stanford University, Groupe Hospitalier Paris Saint-Joseph, Marie Lannelongue Hospital	Cardiac MRI	*Min main PA volume*; *min main PA area*; main PA RAC; mean main PA flow, mean main PA centerline velocity; *mean right PA centerline velocity*; mean systolic main PA spatial average vorticity; mean systolic main PA area fraction of reverse flow; mean main PA fraction of positive helicity; min main PA spatial average HFI; *max right PA*	*mPAP, TPR, % pred RVSEV*	N/A	N/A

			spatial average HFI			
Leong	Hammersmith Hospital	Cardiac MRI	*RV strain*; *RA strain*; *LV-SVI; LV-GLS; peak LA strain; RV-EDVI; RV-ESVI; RV-EF; RV-FWLS; RA-VI; Peak RA strain; LV-EDV/RV-EDV ratio; RV-SV/RV-ESV ratio*	*mPAP; PVR; mRAP*	*REVEAL 2.0 high-risk status score*	N/A
Shahin	University of Sheffield	Cardiac MRI	*LV-SVI; LA-VI*	N/A	*1-year mortality*	N/A
Frederiksen	Aarhus University Hospital	Cardiac MRI	*Change in annular area systole; change in SL distance; change in Tenting height; change in Coaptation height; change in leaflet area systole; change in Tenting volume systole*	*Cardiac output; sPAP; right atrium volume; RV-PA coupling*	N/A	N/A
Czerner	Hannover Medical School Hospital	2D phase-contrast MRI	Acceleration time; acceleration volume; deceleration time; *deceleration volume; max mean velocity; average mean velocity*; max	*mPAP*; PVR; TPR	N/A	*6MWD*

			area; min area; area change			
Pohler	Hannover Medical School Hospital	PREFUL-MRI	*Median pPTT of whole lung; % change in QDPpreful of whole lung; % change of PREFULq of whole lung*	*mPAP*	N/A	*6MWD*
Waziri	Aarhus University Hospital	Cardiac MRI	Change in RA transverse dimension; *change in RV mass*; *change in RV-EF; change in RV-GCS; change in RV-GLS*	*mRAP; sPAP; RV-EF; NT-proBNP*	N/A	N/a
Berman	Papworth Hospital	Cardiac MRI	*Flow per beat; flow per minute; EDV; ESV; RV diastolic mass; RV systolic mass*	*mPAP; PVR; CO; CI*	N/A	6MWD
Reesink	University of Amsterdam, Free University Medical Center, University of California San Diego	Cardiac MRI	*Change in RV-EF; change in RV mass; LVSB*	*TPR*	N/A	N/A

Supplementary Table S2: Studies using imaging predictors for post-PTE outcomes. Italicized predictors indicate the feature reached statistical significance. Italicized outcomes indicate the outcome has a statistically significant imaging predictor [13-16,20,24,25,29–31,41,51–64].

**First Author**	**Centers**	**Imaging Technique**	**Explored Imaging Predictors**	**Hemodynamic Outcomes Predicted**	**Mortality / Morbidity Outcomes Predicted**	**Performance Capacity Outcomes Predicted**
Studies using CT Imaging Predictors						
Koike	Nagasaki University Hospital	DECT	*% change in whole-lung PBV*	*PVR; PAP*; CI; *TRPG; BNP*	N/A	*6MWD*
Tsukada	Keio University School of Medicine, Nihon University School of Medicine	CTPA	*Change in PA diameter; change in RA diameter*; change in dRV/dLV ratio	*mPAP*	N/A	N/A
Zhai	Leiden University Medical Center	CTPA	*Median of density change in vascular areas; IQR of vascular density change*; *median of parenchymal density change; IQR of parenchymal density change*	*mPAP, PVR, sPAP, dPAP, BNP*	N/A	*6MWD*
Ikeda	Tohu University Ohashi Medical Center	Contrast-enhanced CT	*Long axis pulmonary bleeding > 26.5mm; short axis pulmonary bleeding > 17.5mm; 8+ slices*	N/A	*Occurrence of hemoptysis*	N/A

			of visible pulmonary bleeding; bleeding volume > 14.3cm^3			
Ikeda	Toho University Ohashi Medical Center	Non-contrast CT	*Long axis pulmonary bleeding > 31.5mm; short axis pulmonary bleeding > 17.5mm; 7+ slices of visible pulmonary bleeding; bleeding volume > 14.3cm^3*	N/A	*Occurrence of hemoptysis*	N/A
Sugimoto	Fukushima Medical University	Non-contrast CT	*Main PA diameter index; right PA diameter index; left PA diameter index*	N/A	*'Complications' after BPA (asymptomatic lung infiltration and/or hemoptysis)*	N/A
Koike	Nagasaki University Hospital	DECT	*Improvements in lung perfusion blood volume*	*mPAP, PVR,* cardiac index	N/A	*6MWD*
Studies using MRI Predictors						
Yamasaki	Kyushu University	Cardiac MRI	*Change in RA max volume; change in RA min volume; change in RA-EF; change in RA peak longitudinal*	*PVR; BNP*	N/A	6MWD

			strain; change in peak LSR; change in RA early LSR; change in RA late LSR			
Kamada	Tohuku University	4D-flow MRI	Duration of vortical flow at FWHM; area ratio; VFRb	mPAP	N/A	N/A
Roller	University of Giessen	Cardiac MRI	*GLS*	*mPAP; PVR; RV-EF*; AA-T1		
Kawakubo	Kyushu University	Cardiac MRI	*Change in GAS*; change in ASbas; change in ASmid; *change in ASape %*; *change in LS*; change in CS; *change in RS*	mPAP; *PVR; RV-EDVI; RV-ESVI; RV-EF; BNP*	N/A	6MWD
Schoenfeld	Hannover Medical School Hospital	Cardio-pulmonary MRI	*PBF ratio for whole lung; PBF ratio for treated lung; PBF ratio for untreated lung*	CO; ventricular mass index; RV-EF; RV-SVI; FWHM	N/A	6MWD; NYHA functional score
Yamasaki	Kyushu University	Cardiac MRI	LV-EDVI; LV-SVI; RV-EF; RV-EDVI; RV-ESVI	*mPAP*	*Change in interventricular dyssynchrony after BPA*	N/A
Nagao	Tokyo Women's Medical University, Kyushu University	Phase-Contrast MRI	Main PA energy; left PA energy	mPAP	N/A	N/A

Kriechbaum	University of Giessen	Cardiac MRI	*Change in RV-EDV index; change in RV-ESV index; change in RV-EF*	*NT-proBNP; MR-proANP*	N/A	N/A
Nishina	Kyorin University, Keio University	Cardiac MRI	*RV-EDVI*; *RV-ESVI*; RV-SVI; *RV-EF*; *SIR*	*PVR; PVR < 3 Woods units*	N/A	N/A
Studies using Angiography Predictors						
Ikeda*	Tohu University Ohashi Medical Center	Selective pulmonary angiography	Web lesion; ring lesion; abrupt narrowing lesion; *occlusive lesion*	N/A	*Procedure-related 'complications' (pulmonary bleeding events, PA dissections, hematoma, cardiac tamponade); occurrence of hemoptysis*	N/A
Ejiri	Okayama Medical Center	Pulmonary angiography	"BPA-related vascular injury" (findings of contrast extravasation: focal type, stain and pooling type, diffuse blooming type)	N/A	Occurrence of lung injury; use of mechanical ventilator after BPA	N/A
Taniguchi	Universite Paris-Sud (Hopital	DSA	*Poor subpleural perfusion (subpleural spaces*	N/A	*'BPA Failure' – mPAP > 30 mmHg and PVR*	N/A

	Marie Lannelongue, Le Plessis-Robinson, Hopital Bicetre)		*unperfused or minimally perfused in all segments)*		*decrease of < 30% at re-evaluation*	
Maschke	Hannover Medical School Hospital	2D perfusion angiography	*ΔPD of lung parenchyma / PD of inflow ratio; ΔAUC of lung parenchyma / AUC of inflow*; ΔTTP of lung parenchyma / TTP inflow	*'Pulmonary-flow-grade-score' (estimate of lung perfusion from no perfusion, partial segmental artery perfusion, complete segmental artery perfusion, complete segmental artery perfusion, and complete perfusion of pulmonary circulation)*	N/A	N/A
Kawakami	Okayama Medical Center	Selective pulmonary angiography	Ring-like stenosis lesion; web lesion; *subtotal lesion*;	N/A	*% complication rate (balloon injury, wire injury, or vessel*	N/A

			occlusion lesion; tortuous lesion		*dissection) of lesion compared to other types; % success rate (balloon catheter reaching lesion) of lesion compared to other types*	
Kinutani	Kobe University Hospital	Pulmonary angiography	*Pre-BPA minimal lumen diameter*	N/A	*Occurrence of reperfusion pulmonary injury*	N/A
Studies using SPECT Imaging Predictors						
Hashimoto	Toho University Omori Medical Center	SPECT/CT	*Functional % volume of lung*	*mPAP*	N/A	N/A
Maruoka	Kyushu University	SPECT	*Increase in TUV; fractal dimension*	*mPAP*	N/A	N/A

Supplementary Table S3: Studies using imaging predictors for post-BPA outcomes. Italicized predictors indicate the feature reached statistical significance. Italicized outcomes indicate the outcome has a statistically significant imaging predictor [17–23,26–28,32,33,42,65–75].

**Study Characteristics**	**PTE (n=25)**	**BPA (n=23)**	**Total (n=48)**
**Study Design**			
Prospective Cohort, n	4	3	7
Retrospective Cohort, n	21	20	41
**Sample Size**			
Mean	73.4	36.5	55.7
Total, n	1835	839	2674
**Mean Patient Age**	58.4	64.0	60.2
**Unique Imaging Predictors Described, n**	88	64	152
**Mean Follow-up Duration, months**	15.3	3.6	9.7

Supplementary Table S4: Characteristics of included studies by intervention.

1st Author	Journal	Year	Country
Dong	Journal of Cardiovascular Magnetic Resonance	2022	USA, France
Leong	Pulmonary Circulation	2023	UK
Liu	Frontiers in Cardiovascular Medicine	2023	China
Shahin	Frontiers in Medicine	2022	UK
Hashimoto	Journal of Nuclear Cardiology	2022	Japan
Yamasaki	European Heart Journal	2020	Japan
Shikhare	British Journal of Radiology	2022	Singapore/Canada
Kamada	European Journal of Radiology	2022	Japan
Roller	European Journal of Radiology	2022	Germany
Eberhard	Diagnostics	2022	Switzerland/Canada
Frederiksen	BMC Cardiovascular Disorders	2021	Denmark
Koike	Lung	2021	Japan
Tsukada	European Radiology	2021	Japan
Niznansky	Journal of International Medical Research	2021	Czech Republic
McInnis	ERJ Open Research	2020	Canada
Ruigrok	Pulmonary Circulation	2020	Netherlands
Czerner	PLOS ONE	2020	Germany
Pohler	Journal of Magnetic Resonance Imaging	2020	Germany
Waziri	International Journal of Cardiology	2020	Denmark
Kawakubo	European Radiology	2019	Japan
Ikeda	Catheterization and Cardiovascular Interventions	2019	Japan
Schoenfeld	European Radiology	2019	Germany
Saito	American Journal of Respiratory and Critical Care Medicine	2019	Japan
Zhai	Investigative Radiology	2018	Netherlands
Ejiri	Circulation	2018	Japan
Taniguchi	Chest	2018	France
Ikeda	Journal of the American College of Cardiology	2018	Japan
Maruoka	Nuclear Medicine Communications	2017	Japan
Maschke	European Radiology	2017	Germany
Sugimoto	International Heart Journal	2017	Japan
Yamasaki	International Journal of Cardiovascular Imaging	2017	Japan
Nagao	European Journal of Radiology	2017	Japan
Leone	La Radiologia medica	2017	Italy
Kawakami	Circulation: Cardiovascular Interventions	2016	Japan
Koike	European Journal of Radiology	2016	Japan

Tanabe	Chest	2012	Japan
Kunihara	The Annals of Thoracic Surgery	2010	Germany
Shimizu	Circulation	2008	Japan
Oikonomou	Journal of Thoracic Imaging	2004	Canada
Boehm	Journal of Thoracic and Cardiovascular Surgery OPEN	2022	Austria
Kriechbaum	Pulmonary Circulation	2021	Germany
Nishina	Circulation	2020	Japan
Scholzel	The International Journal of Cardiovascular Imaging	2015	Netherlands/Belgium
Kinutani	International Journal of Cardiology	2016	Japan
Berman	Pulmonary Circulation	2014	UK
Reesink	Journal of Thoracic and Cardiovascular Surgery	2007	Amsterdam, USA
Heinrich	Chest	2005	Germany
Bergin	American Journal of Roentgenology	2000	USA

Supplementary Table S5: Country and Journals of Publication [13–24,25–33,41,42,51–75]

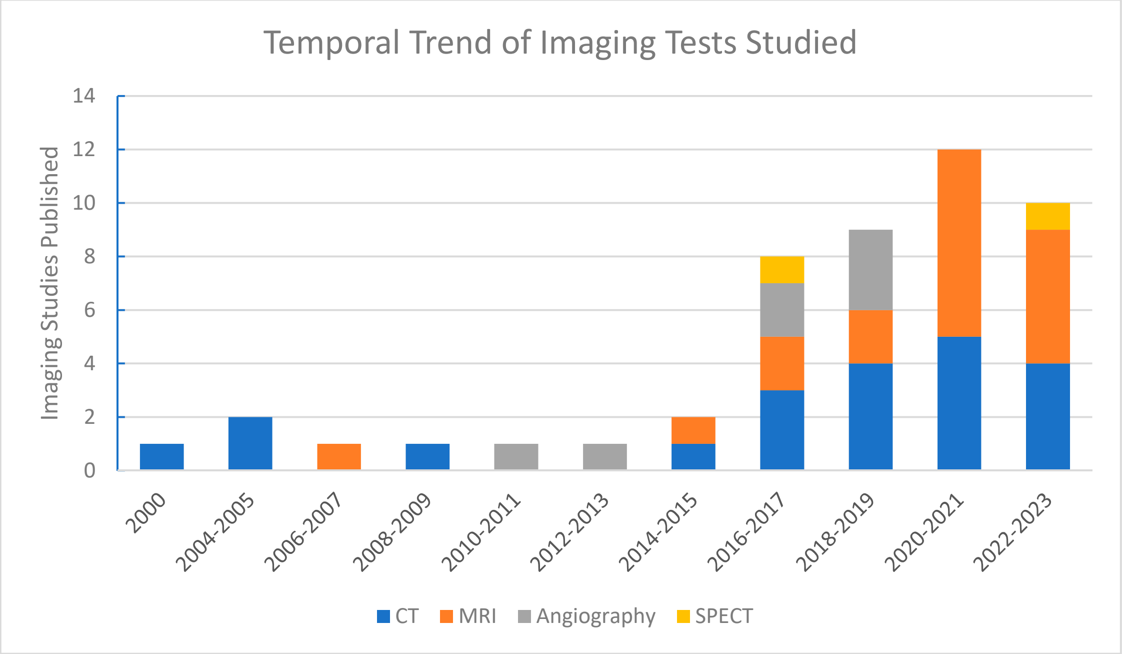

Supplemental Figure S1: Temporal trend of imaging tests studied over time. CT = computed tomography, MRI = magnetic resonance imaging, SPECT = single photon emission computed tomography
